# Large Curvature Self-Folding Method of a Thick Metal Layer for Hinged Origami/Kirigami Stretchable Electronic Devices

**DOI:** 10.3390/mi13060907

**Published:** 2022-06-08

**Authors:** Atsushi Eda, Hiroki Yasuga, Takashi Sato, Yusuke Sato, Kai Suto, Tomohiro Tachi, Eiji Iwase

**Affiliations:** 1 School of Fundamental Science and Engineering, Waseda University, 3-4-1 Okubo, Shinjuku-ku, Tokyo 169-8555, Japan; aeda1996@akane.waseda.jp (A.E.); sato@iwaselab.amech.waseda.ac.jp (T.S.); y-sato@iwaselab.amech.waseda.ac.jp (Y.S.); 2 Faculty of Core Research, Ochanomizu University, 2-1-1 Otsuka, Bunkyo-ku, Tokyo 112-8610, Japan; yasuga.hiroki@ocha.ac.jp; 3 Nature Architects Inc., Akasaka, Minato-ku, Tokyo 107-0052, Japan; kai@nature-architects.com; 4 Graduate School of Arts and Sciences, The University of Tokyo, 3-8-1 Komaba, Meguro-ku, Tokyo 153-8902, Japan; tachi@idea.c.u-tokyo.ac.jp

**Keywords:** self-folding, origami, stretchable device, flexible device

## Abstract

A self-folding method that can fold a thick (~10 μm) metal layer with a large curvature (>1 mm^−1^) and is resistant to repetitive folding deformation is proposed. Given the successful usage of hinged origami/kirigami structures forms in deployable structures, they show strong potential for application in stretchable electronic devices. There are, however, two key difficulties in applying origami/kirigami methods to stretchable electronic devices. The first is that a thick metal layer used as the conductive layer of electronic devices is too hard for self-folding as it is. Secondly, a thick metal layer breaks on repetitive folding deformation at a large curvature. To overcome these difficulties, this paper proposes a self-folding method using hinges on a thick metal layer by applying a meander structure. Such a structure can be folded at a large curvature even by weak driving forces (such as those produced by self-folding) and has mechanical resistance to repetitive folding deformation due to the local torsional deformation of the meander structure. To verify the method, the large curvature self-folding of thick metal layers and their mechanical resistance to repetitive folding deformation is experimentally demonstrated. In addition, an origami/kirigami hybrid stretchable electronic device with light-emitting diodes (LEDs) is fabricated using a double-tiling structure called the perforated extruded Miura-ori.

## 1. Introduction

Stretchable electronic devices have been developed using stretchable materials, such as conductive elastomers and organic semiconductors, or stretchable structures, such as origami-based structures with folds and kirigami structures with slits [1,2,3,4,5,6,7,8,9,10,11,12,13,14,15]. Hinged origami/kirigami stretchable electronic devices consist of panels (undeformed regions) and hinges (local bending deformation regions), and the stretchability of the entire device is achieved by the local bending deformation of the hinges [5,6,7,8,9,10,11,12,13,14,15]. Therefore, the hinged origami/kirigami stretchable electronic devices can consist only of non-stretchable high-performance materials such as rigid electronic elements mounted on the panels and a metal conductive layer on the hinges and panels. Although a metal conductive layer on the hinges should have bendability, a metal conductive layer thickness of at least 10 μm is required for a low electrical resistance of the device due to the thickness of typically printed circuit boards, which is of the order of 10 to 100 μm. In addition, to fabricate a hinged origami/kirigami structure with a panel size of a few millimeters, it is necessary to fold the hinges with a large curvature larger than 1 mm^−1^ by self-folding method. A self-folding is a method to allow a structure to be spontaneously folded by external energy, such as heat or a magnetic field, without human hands directly. Various self-folding methods such as the use of the surface tension of a solder [16,17,18], magnetic materials [19,20,21,22], hydrogel [23,24,25], and heat-shrinkable polymers have been reported [7,26,27,28,29,30,31,32]. There are reports of a self-folding method with large curvature (>1 mm^−1^) and with low bending stiffness materials such as hydrogel or thin metal (<1 µm) [23,24,25,33,34]. However, the bending stiffness of a metal layer thicker than 10 μm is too high to allow hinges to be folded using the self-folding method, namely that the thick metal layer cannot self-fold at a curvature larger than 1 mm^−1^. Even if the driving force of self-folding is sufficient, it is held that a metal layer thicker than 10 μm breaks upon repetitive folding, because the maximum strain on the hinge surface is beyond the elastic range of the metal when it is folded at a curvature larger than 1 mm^−1^ by simple bending deformation. Therefore, we added slits to the hinge to achieve self-folding of the conductive metal layer thicker than 10 μm with a curvature larger than 1 mm^−1^ and mechanical resistance to repetitive deformation.

In this paper, we present the design of the slit structure of the hinge and evaluate the curvature of the self-folding and repetitive deformation resistance. In addition, we demonstrate an origami/kirigami hybrid stretchable electronic device.

## 2. Design and Fabrication

**Figure 1:**
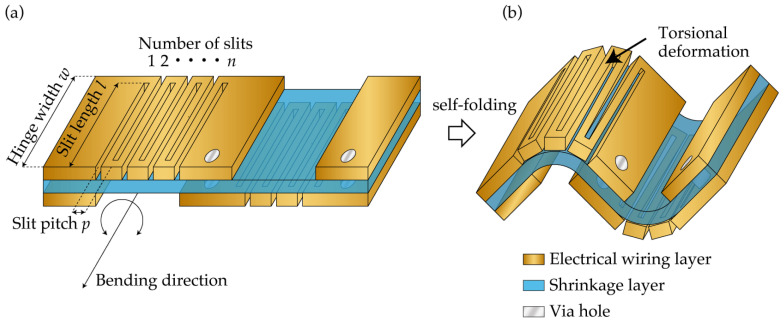
Schematic illustrations of the self-folding method for hinged origami/kirigami stretchable electronic device; (**a**) Structure before folding; (**b**) Structure after folding.

Figure 1 shows the proposed self-folding method for hinged origami/kirigami stretchable electronic devices. The structure before the self-folding is shown in Figure 1a. A heat-shrinkable polymer is sandwiched between two metal layers. The heat-shrinkable polymer and the metal layers constitute the shrinkage layer for self-folding and the electrically conductive layers for an electronic device, respectively. To achieve self-folding with a curvature larger than 1 mm^−1^, the inner metal layer of the fold was removed, and slits were made in the outer metal layer of the fold at the hinges, as shown in Figure 1. By creating the slits in an alternating pattern, hinges with a meander structure can achieve an electrical connection between adjacent panels connected with the mountain fold. Furthermore, through-holes filled with a conductive paste were made in the panels. The through-holes can achieve an electrical connection between adjacent panels connected with the valley fold. Figure 1b shows the structure after the self-folding. We analyzed the effect of the parameters of the meander structure on the apparent bending stiffness of the hinge. We can evaluate the apparent bending stiffness of the hinge with a meander structure by considering that the torsional deformation of the beams of the meander structure results in the self-folding of the thick metal layer, as follows. The hinge width *w*, slit pitch *p*, slit length *l*, and a number of slits *n* were defined as shown in Figure 1a. When the moment generated during the self-folding was *M*, considering the torsional deformation of the beams with a rectangular cross-section, the torsional deformation per beam, *θ*_unit_, is as follows:

(1) $$\theta_{\mathrm{unit}}=\frac{3M(2l\;-\;w)}{{\mathrm{Gt}}^{3}p}$$

where *t* and *G* are the thickness and the transverse modulus of the metal layer, respectively. Here, we assumed that the metal thickness *t* is sufficiently smaller than the slit pitch *p*. Because the number of beams is *n* − 1, the self-folding angle *θ* evaluated using Equation (1) yields:

(2) $${\theta =(n\;-\;1)\theta }_{\mathrm{unit}}=(n\;-\;1)\frac{3M(2l\;-\;w)}{{\mathrm{Gt}}^{3}p}\;.$$

The self-folding curvature *κ* can be defined as:

(3) $$\kappa =\frac{\theta }{(n\;-\;1)p}\;.$$

Using Equations (2) and (3), the self-folding curvature *κ* becomes:

(4) $$\kappa =\frac{3M(2l\;-\;w)}{{\mathrm{Gt}}^{3}p^{2}}\;.$$

From Equation (4), for a hinge with a meander structure, it can be deduced that the apparent bending stiffness can be decreased by decreasing the slit pitch *p* and increasing the slit length *l*, enabling self-folding at large curvatures.

**Figure 2:**
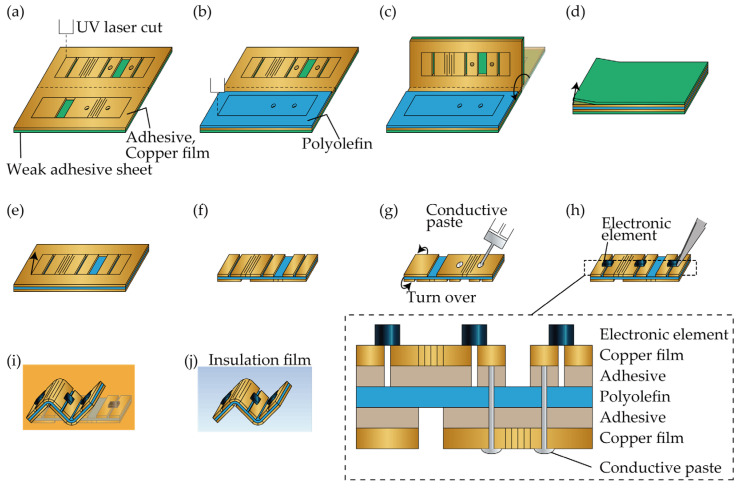
Device fabrication. (**a**) Slitting adhesive and copper film. (**b**) Slitting polyolefin. (**c**) Pasting together. (**d**) Removal of support material. (**e**) Removal of unwanted parts. (**f**) Devices after removal of unwanted parts. (**g**) Filling of the conductive paste. (**h**) Mounting of electronic elements. (**i**) Self-folding. (**j**) Insulating film deposition.

Figure 2 shows the device fabrication method. We used a 10 μm-thick copper film (Nilaco Corporation, Tokyo, Japan, CU-113173) as the metal layer and a 12 μm-thick polyolefin film (Taiyo Electric Ind. Co., Ltd., Tokyo, Japan, HS-2520) as the shrinkage layer, a 5 μm-thick double-sided tape (NEION Film Coatings Corp., Osaka, Japan, Neo Fix 5S2) to bond the layers, a conductive paste (Fujikura Kasei Co., Ltd., Tokyo, Japan, D-723S) as via material, parylene (Specialty Coating Systems Inc., Indianapolis, IN, USA, DPX-C) as an insulating film, and a weak adhesive tape (TECKWRAP, B01MA56OXU) as a support substrate. A UV laser machine (OPI Corporation, Saitama, Japan, OLMUV-355-5A-K), a constant temperature incubator (Yamato Scientific Co., Ltd., Tokyo, Japan, DK300), and a parylene vapor deposition system (Specialty Coating Systems Inc., Indianapolis, IN, USA, PDS2010) were used as the processing equipment. At first, the weak adhesive tape, copper film, and double-sided tape were bonded together in that order from the bottom (Figure 2a) and patterned by the UV laser machine. Two metal layers were patterned on the same sheet, and perforations were made in the center. Then, the polyolefin film was attached to the double-sided tape (Figure 2b) on the side where the electronic devices were not mounted and then patterned using a UV laser machine. The patterned sheet was folded at the central perforation, and the polyolefin film and double-sided tape are bonded together (Figure 2c). Thereafter, the weak adhesive tape was removed (Figure 2d), and the remaining substrate had already been cut off at the outer edge of the hinge structure, so the unwanted parts around it were removed with tweezers (Figure 2e). Figure 2f shows the device without the unwanted parts. Furthermore, the device was turned over, and the through-holes for electrical connections between the layers were filled with conductive paste and then allowed to dry for 24 h (Figure 2g). The electronic elements were then mounted using the conductive paste, as shown in Figure 2h. Furthermore, as shown in Figure 2i, the device was heated at 75 °C for 300 s in a constant temperature incubator to self-fold. The shrink ratio of the polyolefin film at 75 °C is 0.055. If self-folding at high temperature and high shrink ratio is used, the adhesion between the metal and shrinkage layers needs to be increased to prevent exfoliation. Finally, 1 μm-thick parylene was deposited as an insulating film using the parylene vapor deposition system, as shown in Figure 2j.

## 3. Evaluation and Discussion

**Figure 3:**
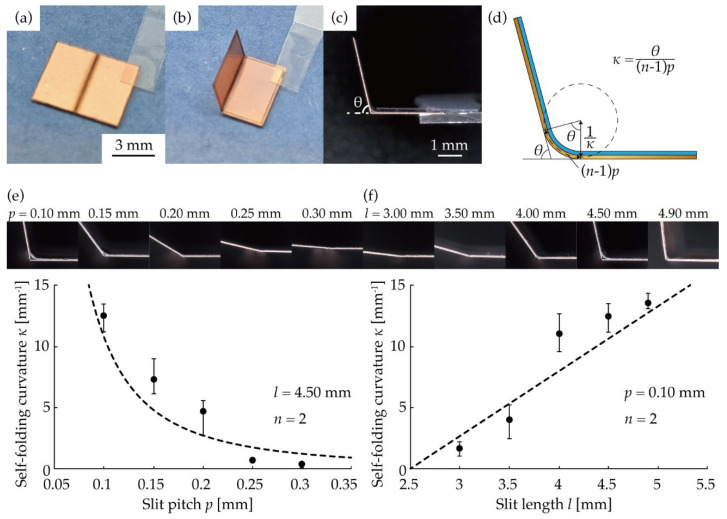
Evaluation of the self-folding curvature. (**a**) Specimen before self-folding. (**b**) Specimen after self-folding. (**c**) Specimen after self-folding observed from the side. (**d**) Measurement method of the self-folding curvature. (**e**) Relationship between the slit pitch *p* and the self-folding curvature *κ*. (Number of slits *n* = 2 and slit length *l* = 4.50 mm.) (**f**) Relationship between the slit length *l* and the self-folding curvature *κ*. (Number of slits *n* = 2 and the slit pitch *p* = 0.10 mm).

Firstly, we evaluated the self-folding curvature. Specimens having one hinge were fabricated, and the self-folding curvature *κ* was measured by varying the parameters of the slits at the hinge. Figure 3a shows the specimen before the self-folding. The hinge has a meander structure with periodicity in the direction orthogonal to the direction of bending. The hinge width *w* was set to 5.00 mm. Figure 3b shows the specimen after the self-folding. As shown in Figure 3c, the specimens were observed from the side, and the supplementary angle between the two panels was measured as the self-folding angle *θ*. The supplementary angle between the two panels is equal to the self-folding angle *θ*, which is the central angle of the sector shape tangent to the two panels (Figure 3d). The self-folding curvature *κ* was calculated from the measured self-folding angle *θ* and Equation (3). The experimental results are shown in Figure 3e,f. Three specimens were measured for each parameter, and the average value was plotted on the graphs, with the maximum and minimum values indicated by error bars. The fitting curves are shown as dotted lines based on Equation (4) and the experimental values. Figure 3e shows the relationship between the slit pitch *p* and the self-folding curvature *κ* when a number of slits at *n* = 2 and the slit length at *l* = 4.50 mm were fixed, and the slit pitch *p* was varied. The self-folding curvature *κ* decreased with an increase in slit pitch *p*. For the slit pitch *p* < 0.20 mm, the self-folding curvature of *κ* > 1 mm^−1^ could be obtained. The maximum value of the self-folding curvature *κ* was 12 mm^−1^ for the slit pitch of *p* = 0.10 mm. The moment *M* was assumed to be constant regardless of the slit pitch *p* and fitted to the experimental data using the least-squares method. In this experiment, the root mean square of the error between the experimental and fitted values of curvature was minimized when *M*/*G* = 8.9 × 10^−18^ m^−1^. Figure 3f shows the relationship between the slit pitch *p* and the self-folding curvature *κ* when the number of slits at *n* = 2 and the slit pitch at *p* = 0.10 mm were fixed and the slit length *l* was varied. The self-folding curvature *κ* increased with an increase in slit length *l*. This is because the torsional deformation in the beam increases with an increase in the slit length. For the slit length of *l* < 3.00 mm, the self-folding curvature *κ* > 1 mm^−1^ could be obtained. The self-folding curvature *κ* reached a maximum value of 14 mm^−1^ at the slit length *l* = 4.90 mm. Here, too, the moment *M* was assumed to be constant, regardless of the slit length *l*, and was fitted to the experimental data using the least-squares method. Similarly, the root mean square of the error between the experimental and fitted values of curvature was minimized when *M*/*G* = 8.9 × 10^−18^ m^−1^.

**Figure 4:**
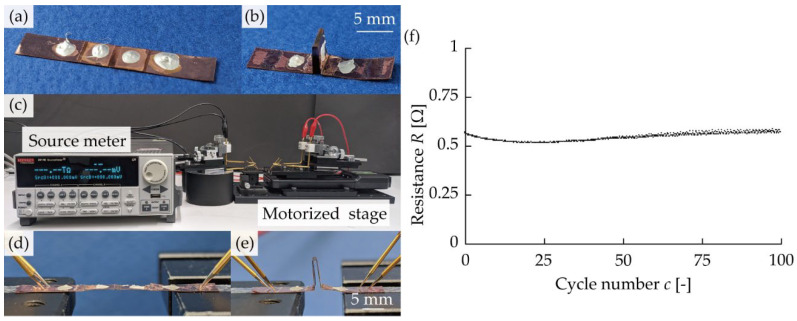
Evaluation of the repetitive deformation resistance. (**a**) Specimen before self-folding. (**b**) Specimen after self-folding. (**c**) Experimental setup. (**d**) Specimen during extension. (**e**) Specimen during contraction. (**f**) Change of the electrical resistance in 100 cycles.

Next, we evaluated mechanical resistance for repetitive deformation. The bellows-folded specimens, shown in Figure 4a,b, were fabricated with a hinge width of 5.00 mm. Three hinges (valley-fold, mountain-fold, and valley-fold from the end) were provided, and the two ends of the four panels were parallel to the direction of the stretching deformation. The length of the panels was 9.85, 4.40, 4.40, and 9.85 mm from the end. For the valley-fold hinge, the slit pitch, slit length, and number of slits were set at *p* = 0.10 mm, *l* = 4.90 mm, and *n* = 4, respectively. For the mountain-fold hinge, the slit pitch, slit length, and number of slits were set at *p* = 0.10 mm, *l* = 4.90 mm, and *n* = 10, respectively. Figure 4c shows the setup for the repetitive deformation test. The specimens were placed onto a motorized stage (Sigma Koki Co., Ltd., Sumida-Ku, Tokyo, SGSP20-85), and the electrical resistance was measured by a source meter (Keithley Instruments, Cleveland, OH, USA, 2614 B) while displacement was applied. The specimen was repeatedly deformed into the states shown in Figure 4d,e, with the folding angle of the mountain-fold at 0° in the first case, and a displacement of 8.80 mm (sum of the lengths of the two panels adjacent to the mountain-fold hinge) applied in the other. The deformation was performed at a frequency of 0.794 Hz, and the electrical resistance was measured by placing probes on the two end panels five times per second using the four-terminal method with a load voltage of 1 mV. Measurements were performed on the three samples, and the results are presented in Figure 4f. At the beginning of the repetitive deformation test, the initial electrical resistance was 0.569 Ω. The rate of change in the electrical resistance was 9.35% after 100 cycles of deformation. At the hinge after repeated deformation, no mechanical damage was observed in the metal layer, and the rate of change in the electrical resistance was small, indicating that there was no electrical damage.

**Figure 5:**
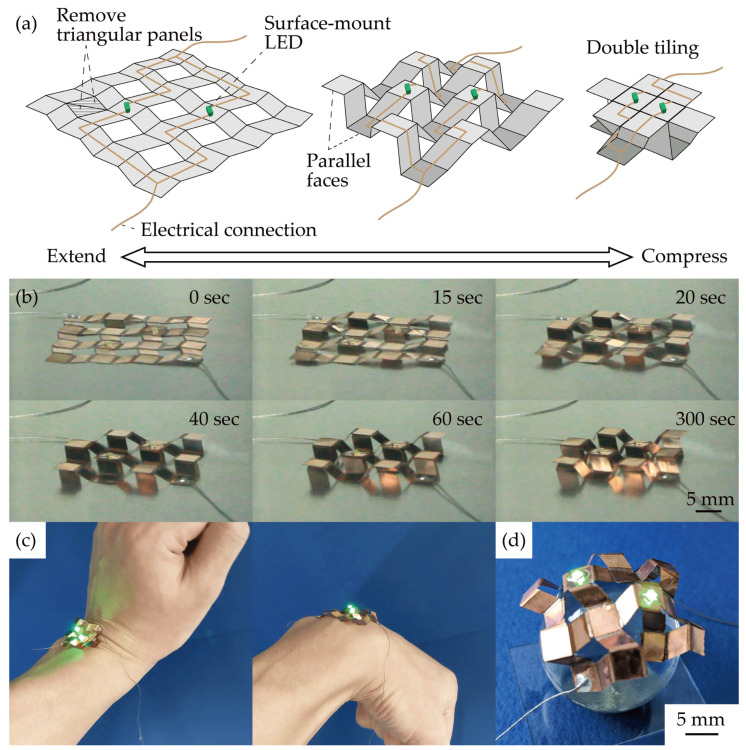
Demonstration. (**a**) Schematic diagram of the device. (**b**) Device during self-folding. (**c**) Attached to a curved human wrist. (**d**) Attached to a 30 mm diameter sphere.

Finally, we fabricated and demonstrated a stretchable electronic device with an origami/kirigami hybrid structure using the proposed self-folding methods. Figure 5a shows schematic illustrations of the device using a “perforated extruded Miura-ori”. An “extruded Miura-ori” structure has parallel faces and a double-tiling structure [35]. The extruded Miura-ori structure with parallel faces is suitable for a stretchable electronic device because the lower faces can be used for attaching curved surfaces, and the upper faces can be used for mounting part of the electronic elements. We used a “perforated extruded Miura-ori” structure, which is a structure with triangular panels removed from an extruded Miura-ori structure, as shown in Figure 5a. The perforated extruded Miura-ori is suitable for self-folding because it prevents the concentration of lines at the vertices and is suitable for attaching curved surfaces, as it increases the degree of freedom by removing the triangular panels. Surface-mounted LED chips (Stanley Electric Co., Ltd., Tokyo, Japan, VCDG1112H-4BY3C-TR) were mounted on the device and emitted by applying a voltage of 3 V. Figure 5b shows the self-folding behavior of the device. Each hinge gradually folds tightly with time. Figure 5c shows the device attached to a human wrist. The LEDs could continue to emit even after 100 cycles of stretching and contraction. Furthermore, the LEDs could be emitted on a sphere of 30 mm in diameter (Figure 5d). These results indicate that the proposed structure and self-folding methods can be used as a stretchable electronic device.

## 4. Conclusions

In this paper, we present the design of a hinge structure for a self-folding thick metal layer with a large curvature and mechanical resistance to repetitive deformation. This is achieved by applying a meander structure to the hinge and using the torsional deformation of the beams of the meander structure. The experiments demonstrated a large curvature self-folding of up to 14 mm^−1^ with a metal layer of 10 μm in thickness. We also confirmed that the device could withstand more than 100 repetitive deformations. Finally, we fabricated an origami/kirigami hybrid structure with LEDs and demonstrated that the structure can be used as a stretchable electronic device. This paper enables the fabrication of hinged origami/kirigami stretchable electronic devices using thick and non-stretchable high-performance materials.
